# Leukemogenic SHP2 mutations lead to erythropoietin independency of HCD-57 cells: a novel model for preclinical research of SHP2-mutant JMML

**DOI:** 10.1186/s40164-023-00379-1

**Published:** 2023-02-20

**Authors:** Yuming Zhao, Chunxiao He, Dengyang Zhang, Yao Guo, Zhiyong Peng, Liuting Yu, Na Li, Chun Chen, Zhizhuang Joe Zhao, Yun Chen

**Affiliations:** 1 Department of Pediatrics, Edmond H. Fischer Translational Medical Research Laboratory, Scientific Research Center, The Seventh Affiliated Hospital, Sun Yat-Sen University, Shenzhen, 518107 Guangdong China; 2 Nanfang-Chunfu Children’s Institute of Hematology, Taixin Hospital, Dongguan, Guangdong China; 3 Department of Pediatrics, The Seventh Affiliated Hospital, Sun Yat-Sen University, Shenzhen, 518107 Guangdong China; 4 Department of Pathology, University of Oklahoma Health Sciences Center, 1100 N. Lindsay, Oklahoma City, OK 73104 USA

**Keywords:** SHP2, Cell model, JMML, HCD-57

## Abstract

Leukemogenic SHP2 mutations occur in 35% of patients with juvenile myelomonocytic leukemia (JMML), a rare but fatal hematopoietic malignancy without representative cell models, which are urgently needed to investigate the pathogenesis and to develop novel therapeutic strategies. In this study, we established stable cell lines with aberrant signaling resembling SHP2-mutant JMML through retroviral expression of SHP2-D61Y/E76K in HCD-57 cells, a murine erythroleukemia cell line that depends on erythropoietin (EPO) for survival. SHP2-D61Y/E76K drives the survival and proliferation of HCD-57 cells in the absence of EPO, but not in Ba/F3 cells in the absence of IL-3. Transformed HCD-57 cells showed activated MAPK signaling that is consistent with SHP2-mutant JMML. Transformed HCD-57 cells were sensitive to dasatinib and trametinib, two targeted drugs previously reported to inhibit SHP2-mutant JMML cells. Furthermore, we injected mutant SHP2-transformed HCD-57 cells into immune-deficient mice intravenously and found that these cells rapidly proliferated in the spleen and bone marrow, providing an excellent model for in vivo testing of drugs targeting the aberrant signaling of mutant SHP2. In conclusion, we established the novel cell lines HCD-57/SHP2-E76K and -D61Y that depended on signaling of mutant SHP2 for survival, thus resembling SHP2-mutant JMML. Our model is a valuable tool to investigate the pathogenic mechanisms of mutant SHP2 and targeted drugs for SHP2-mutant JMML.

**Supplementary Information:**

The online version contains supplementary material available at 10.1186/s40164-023-00379-1.

**To the editor,**

SHP2 is a nonreceptor protein tyrosine phosphatase (PTP) that plays key roles in many cytokine/growth factor-dependent signaling pathways [1]. The catalytic activity of SHP2 is autoinhibited by the N-terminal SH2 (N-SH2) domain. Therefore, mutations disrupting the autoinhibitory function of N-SH2 constitutively activate SHP2, leading to hematopoietic malignancies and several genetic disorders [2]. Leukemogenic SHP2 mutations occur in ~ 35% of patients with juvenile myelomonocytic leukemia (JMML), a rare but highly fatal myeloproliferative neoplasm [3]. Previous studies have found that patients with mutant SHP2 had an adverse prognosis compared with other genetic subtypes [4]. In general, the outcomes of JMML remain poor with conventional chemotherapy, and novel therapeutic strategies are urgently needed [5]. Currently, studies on targeted therapies for JMML are limited by the rarity of the disease and the lack of suitable cell models. The IL-3-dependent pro-B-cell line Ba/F3 is the most popular system to generate models with oncogene dependency and vulnerability to targeted drugs [6]. However, SHP2 mutants in JMML failed to transform Ba/F3 cells. Since Ba/F3 is a lymphoid cell line, we speculate that cells with myeloid features could be potentially transformed by leukemogenic SHP2 mutants. Previously, we generated models representing FLT3 and c-KIT-mutant malignancies with HCD-57 cells, a murine myeloid erythroleukemia cell line that depends on erythropoietin (EPO) for survival [7, 8]. Here, we explored HCD-57 as a novel model to represent SHP2-mutant JMML.

We generated HCD-57 cells expressing SHP2-D61Y or -E76K by using the MSCV-IRES-EGFP retrovirus expression vector. These GFP-positive cells expanded in the absence of EPO, but parental HCD-57 cells and cells expressing wild-type SHP2 did not (Fig. 1A). Flow cytometry showed that HCD-57/SHP2-D61Y or -E76K had a negligible amount of apoptotic cells after EPO withdrawal. In contrast, deprivation of EPO resulted in significant amounts of apoptotic cells in control cells (Fig. 1B, C). We also analyzed Ba/F3 cells expressing wild-type or mutant SHP2. Flow cytometry showed that Ba/F3 cells expressing wild-type or mutant SHP2 had similar levels of apoptosis after withdrawal of IL-3 (Fig. 1D, E). In addition, we found that the expression of SHP2-D61Y or -E76K led to significantly increased levels of ERK phosphorylation in HCD-57 cells compared with control cells (Fig. 1F). Transcriptome analysis showed that 585 upregulated genes shared by HCD-57/SHP2-D61Y and -E76K compared with parental cells were significantly involved in the MAPK signaling pathway by using Kyoto Encyclopedia of Genes and Genomes (KEGG) analysis (Fig. 1G, H). Furthermore, gene set enrichment analysis (GSEA) revealed that the MAPK pathway of HCD-57 cells expressing SHP2-D61Y or SHP2-E76K was significantly upregulated compared with that in parental cells (Fig. 1I). Several key genes involved in the MAPK pathway were notably upregulated in SHP2-mutant cells, including *Map3k7*, *Dusp6* and *Atf4* (Fig. 1J). Hypersensitivity to granulocyte macrophage-colony-stimulating factor (GM-CSF) is a feature of JMML cells. However, HCD-57/SHP2-D61Y and -E76K did not respond to GM-CSF stimulation (Additional file 1: Fig. S1). We found that HCD-57 cells had negligible expression of receptors for GM-CSF (Additional file 1: Table S1), which is a different feature between our model and primary JMML cells. Our data suggested that the dependency of JMML cells on SHP2 mutants may not require GM-CSF signaling, which needs further investigation. Recently, induced pluripotent stem cells (iPSCs) generated from primary cells from patients with JMML have provided a valuable cell source for investigating JMML biology [9]. However, iPSCs are inconvenient and expensive. Additionally, the heterogeneity of the disease may interfere with the use of iPSCs to study leukemogenic mechanisms and drug screening. Here, we provided a convenient and clean cell system that depends on leukemogenic SHP2 mutations for survival, along with parental HCD-57 cells as controls, which are useful tools to study JMML in parallel with iPSCs and primary cells.

**Fig. 1:**
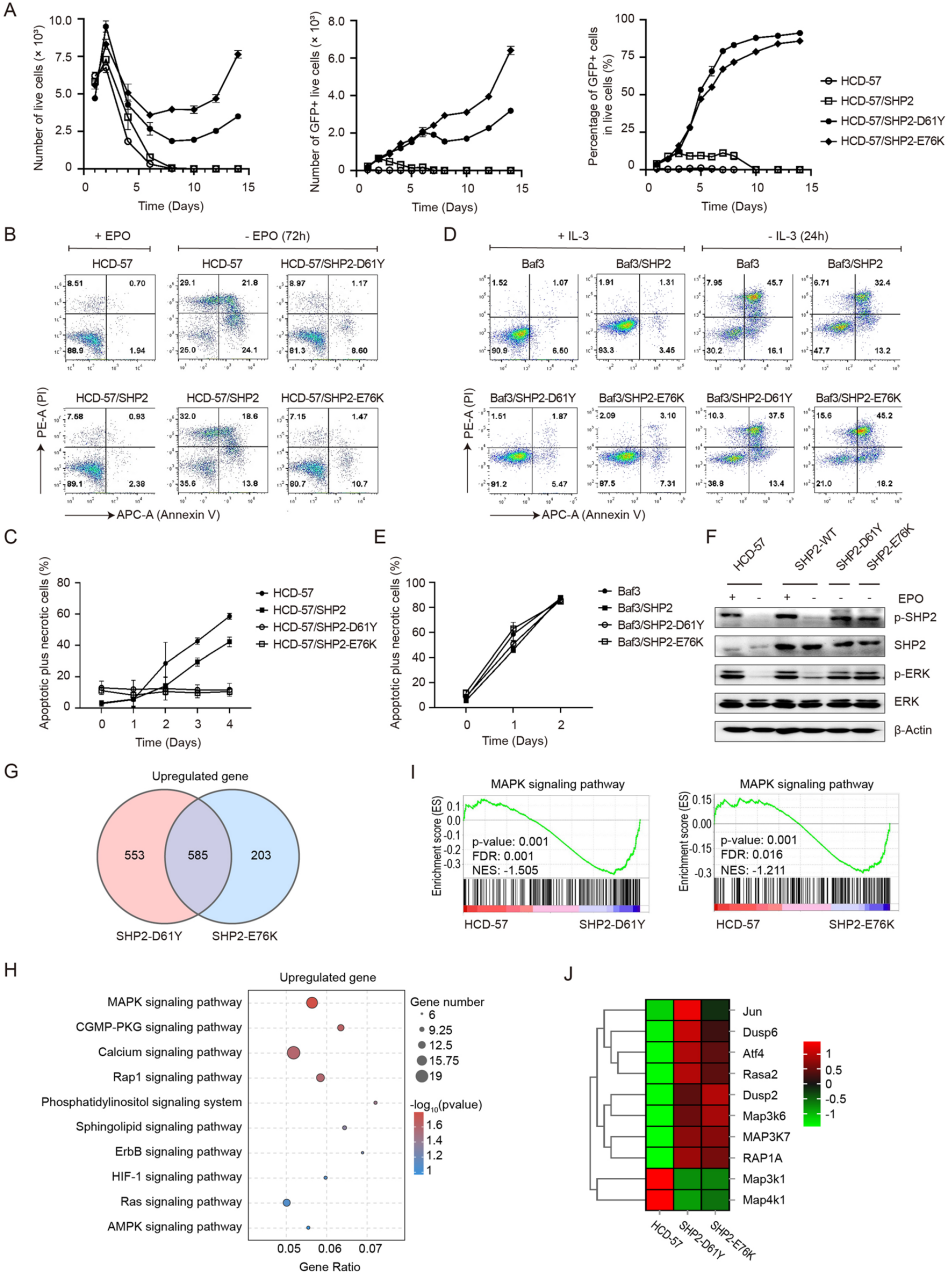
Expression of SHP2-D61Y and -E76K leads to cytokine-independent survival and proliferation in HCD-57 cells with the activation of the MAPK signaling pathway. **A** Number of live cells, GFP-positive cells, and the percentage of GFP-positive cells in live cells analyzed by flow cytometry of HCD-57 cells expressing SHP2-D61Y, SHP2-E76K or wild-type SHP2 in the absence of EPO. **B**, **C** Percentage of apoptotic cells (Annexin V-positive) in sorted GFP + HCD-57 cells cultured with or without EPO. **D**, **E** Percentage of apoptotic cells (Annexin V-positive) in sorted GFP + Ba/F3 cells cultured with or without IL-3. The error bar denotes the standard deviation. **F** Immunoblot analysis of pSHP2 (Tyr542), SHP2, pERK (Tyr202/204) and ERK in parental HCD-57 cells and HCD-57 cells expressing SHP2, SHP2-D61Y or E76K cultured with or without EPO. **G** Venn diagram indicating the overlapping upregulated genes in HCD-57 vs*.* SHP2-D61Y and HCD-57 vs*.* SHP2-E76K. **H** KEGG analysis of the shared upregulated genes in HCD-57 cells expressing SHP2-D61Y or -E76K compared with parental cells. The signal transduction pathways are summarized. **I** GSEA plots of MAPK signaling pathway target genes in HCD-57 cells expressing SHP2-D61Y or -E76K *vs.* parental cells. Normalized ES (NES), nominal p value and FDR q-values are indicated. **J** Heatmap of representative differentially expressed genes involved in the MAPK signaling pathway

To validate our system for use in drug discovery for SHP2-mutant JMML, we screened 16 clinically available kinase inhibitors by using our model. We identified dasatinib and trametinib as potent inhibitors targeting HCD-57 cells expressing mutant SHP2 (Fig. 2A). The cell viability assay demonstrated that dasatinib and trametinib inhibited cells expressing mutant SHP2 in a dose-dependent manner (Fig. 2B). Immunoblotting showed that dasatinib and trametinib blocked the activation of ERK. In addition, dasatinib but not trametinib inhibited the phosphorylation level of AKT (Fig. 2C, D). To assess the engraftment potential of mutant SHP2-transformed HCD-57 cells in vivo, we intravenously injected immune-deficient mice with HCD-57/SHP2-D61Y cells. We found that the weights of spleens in the engrafted group were significantly larger than those in the vehicle group (Fig. 2E). The implanted HCD-57/SHP2-D61Y cells occupied ~ 60% and ~ 90% of the bone marrow and spleen, respectively, as revealed by the proportion of GFP-positive cells analyzed by flow cytometry (Fig. 2F, G). The use of primary cells and iPSCs leads to the identification of several targeted drugs that inhibit JMML cells. Trametinib, which targets RAS signaling pathway, is currently under clinical evaluation for patients with JMML (NCT03190915). The SRC and BCR-ABL inhibitor dasatinib was also effective to inhibit JMML cells in a preclinical study [10]. Our small-scale screening also identified trametinib and dasatinib as potent inhibitors targeting SHP2-mutant HCD-57 cells, indicating the effectiveness of our system. Additionally, the SHP2 allosteric inhibitors RMC-4550 and SHP099 failed to target HCD-57/SHP2-D61Y/E76K (Additional file 1: Fig. S2), which was consistent with previous studies [11, 12]. A large-scale screening may lead to the identification of more effective drugs targeting SHP2-mutant JMML, which may find important clinical applications in the future. Our study not only shows that gain-of-function mutations of SHP2 are fully capable of transforming cytokine-dependent cells but also provides a unique cell model to study the pathogenesis of SHP2 mutants and to identify targeted drugs for JMML, as well as other hematopoietic malignancies driven by SHP2 mutants.

**Fig. 2:**
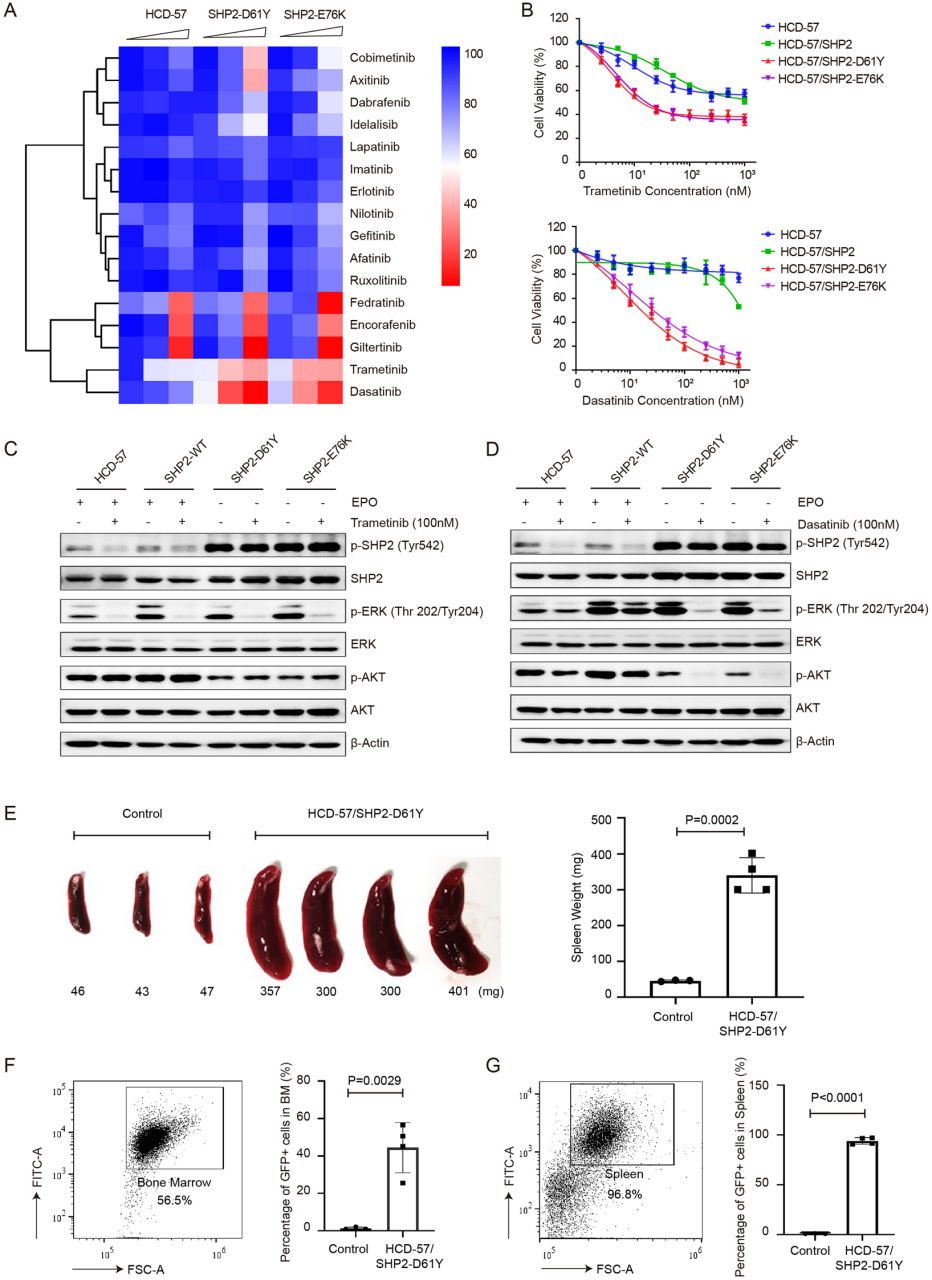
HCD-57 cells expressing mutant SHP2 are effective tools for drug screening and xenografted mouse models. **A** Drug screening by using HCD-57 expressing mutant SHP2 and parental cells with a library containing 16 kinase inhibitors. **B** Cell viability of HCD-57 cells expressing mutant SHP2 and parental cells treated with various concentrations of dasatinib and trametinib. **C**, **D** Immunoblot of pSHP2 (Tyr542), SHP2, pERK (Tyr202/204), ERK, pAKT (Ser473), and AKT in HCD-57 cells expressing mutant SHP2 and parental cells. **E** Weights of spleens of immune-deficient mice injected with HCD-57 cells expressing SHP2-D61Y. **F**, **G** Flow cytometry analysis of cells from the bone marrow and spleen of mice injected with HCD-57 cells expressing SHP2-D61Y, which are positive for GFP. The error bar denotes the standard deviation

## Supplementary Information

**Additional file 1: Figure S1**. The response of HCD-57 to stimulation of GM-CSF. (A) Parental HCD-57 and HCD-57 expressing wild-type SHP2, SHP2-D61Y, and SHP2-E76K were stimulated by increasing concentrations of GM-CSF for 72 hours and the cell viability was assessed by CCK-8. (B) Immunoblotting analysis of p-SHP2 and p-ERK in parental HCD-57 and HCD-57 expressing wild-type SHP2, SHP2-D61Y, and SHP2-E76K stimulated by GM-CSF for 10 minutes. **Figure S2**. Cell viability of HCD-57 expressing mutant SHP2 and parental cells treated by various concentrations of SHP099 or RMC-4550. HCD-57 was cultured in medium with EPO and HCD-57/SHP2-D61Y or -E76K cells were cultured without EPO. Cells were incubated with inhibitors for 48 hours the cell viability was assessed by CCK-8. **Table S1**. The FPKM values of specific genes detected by RNA-Seq.

## Data Availability

All data generated or analyzed during this study are included in this published article.

## Abbreviations

JMML: Juvenile myelomonocytic leukemia
EPO: Erythropoietin
PTP: Protein tyrosine phosphatase
N-SH2: N-terminal Src homology 2
KEGG: Kyoto Encyclopedia of Genes and Genomes
GSEA: Gene set enrichment analysis
GM-CSF: Granulocyte macrophage-colony-stimulating factor
iPSCs: Induced pluripotent stem cells
