# Supplementary material for: Leukemogenic SHP2 mutations lead to erythropoietin independency of HCD-57 cells: a novel model for preclinical research of SHP2-mutant JMML
Source: Exp Hematol Oncol. 2023 Feb 20;12:20. doi: 10.1186/s40164-023-00379-1 (PMC9940371; doi:10.1186/s40164-023-00379-1)
Supplement: Supplementary file 1 — Additional file 1: Figure S1. The response of HCD-57 to stimulation of GM-CSF. (A) Parental HCD-57 and HCD-57 expressing wild-type SHP2, SHP2-D61Y, and SHP2-E76K were stimulated by increasing concentrations of GM-CSF for 72 hours and the cell viability was assessed by CCK-8. (B) Immunoblotting analysis of p-SHP2 and p-ERK in parental HCD-57 and HCD-57 expressing wild-type SHP2, SHP2-D61Y, and SHP2-E76K stimulated by GM-CSF for 10 minutes. Figure S2. Cell viability of HCD-57 expressing mutant SHP2 and parental cells treated by various concentrations of SHP099 or RMC-4550. HCD-57 was cultured in medium with EPO and HCD-57/SHP2-D61Y or -E76K cells were cultured without EPO. Cells were incubated with inhibitors for 48 hours the cell viability was assessed by CCK-8. Table S1. The FPKM values of specific genes detected by RNA-Seq. [file 40164_2023_379_MOESM1_ESM.docx]

**SUPPLEMENTAL INFORMATION**

**Leukemogenic SHP2 Mutations Lead to Erythropoietin Independency of HCD-57 Cells: A Novel Model for Preclinical Research of SHP2-Mutant JMML**

Yuming Zhao^1*^, Chunxiao He^1*^, Dengyang Zhang^1*^, Yao Guo^1^, Zhiyong Peng^2^, Liuting Yu^1^, Na Li^1^, Chun Chen^3#^, Zhizhuang Joe Zhao^4#^, Yun Chen^1#^

^1^Edmond H. Fischer Translational Medical Research Laboratory, Scientific Research Center, The Seventh Affiliated Hospital, Sun Yat-sen University, Shenzhen, Guangdong, China.

^2^Nanfang-Chunfu Children's Institute of Hematology, Taixin Hospital, Dongguan, Guangdong, China

^3^Department of Pediatrics, The Seventh Affiliated Hospital, Sun Yat-sen University, Shenzhen, Guangdong, China.

^4^Department of Pathology, University of Oklahoma Health Sciences Center, 1100 N. Lindsay, Oklahoma City, OK 73104, USA.

^#^Correspondence: Dr. Yun Chen, Department of Pediatrics, Edmond H. Fischer Translational Medical Research Laboratory, The Seventh Affiliated Hospital, Sun Yat-sen University, Shenzhen, 518107 Guangdong, China; phone: (0755)81207021; email: cheny653@mail.sysu.edu.cn

Dr. Zhizhuang Joe Zhao, Department of Pathology, University of Oklahoma Health Sciences Center, Oklahoma City, Oklahoma 73104, USA; phone: 405-271-9344; email: joe-zhao@ouhsc.edu

Dr. Chun Chen, Department of Pediatrics, The Seventh Affiliated Hospital, Sun Yat-sen University, Shenzhen, 518107, Guangdong, China. phone: (0755)81206752; email: chenchun@mail.sysu.edu.cn;

^*^These authors contributed equally to this work.

**Running title:** HCD-57: A Novel Model for JMML

**Methods and materials**

*Reagents*

Murine EPO and IL-3 were from Peprotech (NJ, USA). Murine GM-CSF was from Sino Biological (Beijing, China). Kinase Inhibitors, SHP099, and RMC-4550 were from Selleckchem (TX, USA). All inhibitors were formulated in dimethyl sulfoxide (DMSO) for *in vitro* cell-based assays. CCK-8 was from Solarbio (Beijing, China). Antibodies against pSHP2 (Tyr542) (#3751), SHP2 (#3397), pERK (Thr202/Tyr204) (#4370), ERK (#4695), pAKT (Ser473) (#4060), AKT (#4691), β-Actin (#5125) and secondary antibody conjugated with horseradish peroxidase (#7074) were from Cell Signaling Technology (MA, USA). 7-AAD and Annexin V was from BD Pharmingen (CA, USA).

*Cell culture*

HCD-57 cells were maintained in IMDM (Iscove's modified Dulbecco's medium) containing 20% fetal bovine serum (FBS) plus 20 ng/mL EPO, and Ba/F3 cells were cultured in RPMI with 10% FBS and 1 ng/mL IL-3. All cells were cultured in a humidified atmosphere at 37 °C with 5% CO_2_.

*Generation of mutant SHP2-transformed HCD-57 cells*

Retroviruses expressing mutant SHP2 were generated by using the pMSCV-IRES-GFP vector as described previously.(1, 2) Briefly, pMSCV-IRES-GFP containing the full-length forms of SHP2, SHP2-D61Y, and SHP2-E76K were used to transfect GP2-293 cells together with pVSV-G helper plasmid. Recombinant retroviruses were isolated by centrifugation at 20,000 g for 2 hours and then used to infect HCD-57 cells in the presence of 5 μg/ml polybrene (Merck, USA) under centrifugation at 1800 g for 2 hours at room temperature. Infected HCD-57 cells were cultured in IMDM with 20% FBS in the presence of EPO for 24 hours and then seeded in a semisolid medium containing IMDM, 20% FBS and 1% methylcellulose in the absence of EPO. Ba/F3 cells were infected by retroviruses carrying mutant SHP2 with the same method.

*Immunoblotting*

Immunoblotting was performed as described previously.(3-5) Briefly, total protein was extracted with a whole-cell extraction buffer containing 25 mM β-glycerophosphate (pH 7.3), 5 mM EDTA, 2 mM EGTA, 5 mM β-mercaptoethanol, 1% TritonX-100, 0.1 M NaCl, 1 mM sodium vanadate. Cell lysates were cleared by centrifugation at 12000 g. Equal amounts of total proteins were electrophoresed in a 10% SDS-PAGE gel and transferred to PVDF membranes for blotting analyses with antibodies against pSHP2 (Tyr542), SHP2, pERK (Thr202/Tyr204), ERK, pAKT (Ser473), AKT, and β-Actin.

*Cell viability and apoptosis assays*

Cells were seeded into 96-well plates at 2×10^4^ cells/well and incubated with different concentrations of inhibitors for 48 hours. Stock solutions of inhibitors were made in DMSO and the final concentration of DMSO in the cell incubation system was controlled at 0.1%. For cell viability assay, 10 μL CCK-8 was added into each well, and absorbance at 450 nm was measured after 3 hours incubation at 37 °C. Flow cytometry and apoptosis assay were performed as described previously.(5, 6) Flow cytometric analyses were performed by using Cytoflex Flow cytometer (Backman, USA). Flow data were analyzed by FlowJo VX (OR, USA).

*Transcriptome analysis*

RNA-seq and Gene Set Enrichment Analysis were according to the previous study.(7) HCD-57, HCD-57/SHP2-D61Y, and HCD-57/SHP2-E76K were subjected into RNA-seq analysis with single sample in each group. Total RNA was extracted by using TRIzol (Thermo Fisher Scientific, USA). Next generation sequencing and data analysis were performed by Beijing Genomics Institute (Beijing, China). GSEA was performed by using GSEA software (Version: 4.1.0) on primary RNA-seq data from parental HCD-57 and HCD-57 cells expressing SHP2-D61Y or -E76K. The annotated gene set file (c2.cp.kegg.v7.5.symbols.gmt) was used for the analysis.(8) Normalized enrichment score (NES) and false discovery rate (FDR) were used to quantify enrichment magnitude and statistical significance, respectively. Terms with false discovery rate (FDR) <25% and nominal P value <5% were considered to be significantly different.

*In vivo mouse models*

NCG (NOD/ShiLtJGpt-Prkdc*^em26Cd52^*Il2rg*^em26Cd22^*/Gpt) mice were purchased from GemPharmatech (Jiangsu, China). 1×10^6^ HCD-57/SHP2-D61Y cells or vehicle were injected intravenously. The mice were sacrificed at 3 weeks following engraftment and weighed spleen. GFP-positive cells in spleen and bone marrow were measured by flow cytometry. This study was carried out under an approved protocol in accordance with the guide for the care and use of laboratory animals of the Seventh Affiliated Hospital of Sun Yat-sen University.

*Statistical analysis*

Data were shown with means ± SD and analyzed with GraphPad Priam 8.0 software (GraphPad, USA). A two-tailed students’ t-test was performed to compare differences between the groups. p <0 .05 was considered statistically significant.

**Supplementary figures**

Figure S1


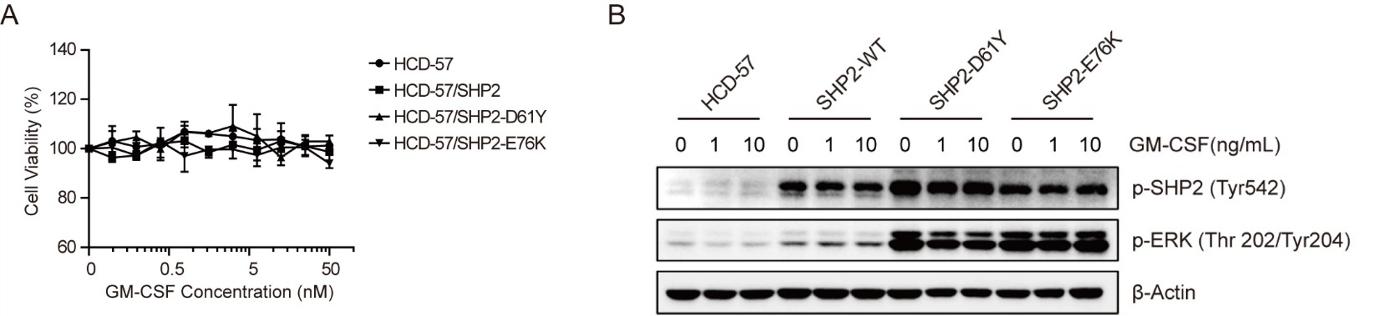


Figure S2


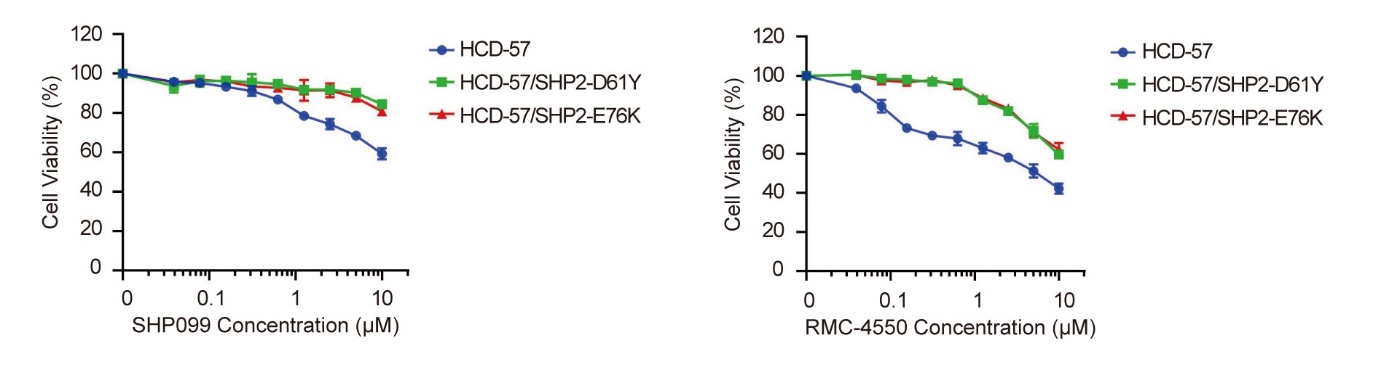


**Supplementary tables**

Table S1. The FPKM values of specific genes detected by RNA-Seq

| Symbol | Description | HCD-57 | SHP2-D61Y | SHP2-E76K |
| --- | --- | --- | --- | --- |
| *Csf2ra* | Colony stimulating factor 2 receptor, alpha, low-affinity (granulocyte-macrophage) | 0 | 0 | 0 |
| *Csf2rb* | Colony stimulating factor 2 receptor, beta, low-affinity (granulocyte-macrophage) | 1 | 1.283 | 0.762 |
| *Csf2rb2* | Colony stimulating factor 2 receptor, beta 2, low-affinity (granulocyte-macrophage) | 0.076 | 0.092 | 0.049 |
| *Epor* | Erythropoietin receptor | 146.177 | 118.652 | 109.257 |

**Supplementary figure legends**

Figure S1. The response of HCD-57 to stimulation of GM-CSF. (A) Parental HCD-57 and HCD-57 expressing wild-type SHP2, SHP2-D61Y, and SHP2-E76K were stimulated by increasing concentrations of GM-CSF for 72 hours and the cell viability was assessed by CCK-8. (B) Immunoblotting analysis of p-SHP2 and p-ERK in parental HCD-57 and HCD-57 expressing wild-type SHP2, SHP2-D61Y, and SHP2-E76K stimulated by GM-CSF for 10 minutes.

Figure S2. Cell viability of HCD-57 expressing mutant SHP2 and parental cells treated by various concentrations of SHP099 or RMC-4550. HCD-57 was cultured in medium with EPO and HCD-57/SHP2-D61Y or -E76K cells were cultured without EPO. Cells were incubated with inhibitors for 48 hours the cell viability was assessed by CCK-8.

**Reference:**

1. Chen Y, Guo Y, Zhao W, Tina Ho WT, Fu X, Zhao ZJ. Identification of an orally available compound with potent and broad FLT3 inhibition activity. Oncogene. 2016;35(23):2971-8.

2. Jacobs-Helber SM, Roh KH, Bailey D, Dessypris EN, Ryan JJ, Chen J, et al. Tumor necrosis factor-alpha expressed constitutively in erythroid cells or induced by erythropoietin has negative and stimulatory roles in normal erythropoiesis and erythroleukemia. Blood. 2003;101(2):524-31.

3. Zhang D, Guo Y, Zhao Y, Yu L, Chang Z, Pei H, et al. Expression of a recombinant FLT3 ligand and its emtansine conjugate as a therapeutic candidate against acute myeloid leukemia cells with FLT3 expression. Microb Cell Fact. 2021;20(1):67.

4. Chen Y, Guo Y, Han J, Ho WT, Li S, Fu X, et al. Generation and characterization of a highly effective protein substrate for analysis of FLT3 activity. J Hematol Oncol. 2012;5:39.

5. Guo Y, Chen Y, Xu X, Fu X, Zhao ZJ. SU11652 Inhibits tyrosine kinase activity of FLT3 and growth of MV-4-11 cells. J Hematol Oncol. 2012;5:72.

6. Guo Y, Pei H, Lu B, Zhang D, Zhao Y, Wu F, et al. Aberrantly expressed Wnt5a in nurse-like cells drives resistance to Venetoclax in chronic lymphocytic leukemia. Cell Death Discov. 2022;8(1):82.

7. Chen Y, Chen L, Yu J, Ghia EM, Choi MY, Zhang L, et al. Cirmtuzumab blocks Wnt5a/ROR1 stimulation of NF-kappaB to repress autocrine STAT3 activation in chronic lymphocytic leukemia. Blood. 2019;134(13):1084-94.

8. Subramanian A, Tamayo P, Mootha VK, Mukherjee S, Ebert BL, Gillette MA, et al. Gene set enrichment analysis: a knowledge-based approach for interpreting genome-wide expression profiles. Proc Natl Acad Sci U S A. 2005;102(43):15545-50.
